# Survival Predictors for Severe ARDS Patients Treated with Extracorporeal Membrane Oxygenation: A Retrospective Study in China

**DOI:** 10.1371/journal.pone.0158061

**Published:** 2016-06-23

**Authors:** Xiaoqing Liu, Yonghao Xu, Rong Zhang, Yongbo Huang, Weiqun He, Ling Sang, Sibei Chen, Lingbo Nong, Xi Li, Pu Mao, Yimin Li

**Affiliations:** The State Key Laboratory of Respiratory Diseases, Guangzhou Institute of Respiratory Diseases, The First Affiliated Hospital of Guangzhou Medical University, Guangzhou Medical University, Guangzhou 510120, China; University of Bari, ITALY

## Abstract

Extracorporeal membrane oxygenation (ECMO) is increasingly being applied as life support for acute respiratory distress syndrome (ARDS) patients. However, the outcomes of this procedure have not yet been characterized in severe ARDS patients. The aim of this study was to evaluate the outcomes of severe ARDS patients supported with ECMO and to identify potential predictors of mortality in these patients. A total of 38 severe ARDS patients (aged 51.39±13.27 years, 32 males) who were treated with ECMO in the specialized medical intensive care unit of Guangzhou Institute of Respiratory Diseases from July 2009 to December 2014 were retrospectively reviewed. The clinical data of the patients on the day before ECMO initiation, on the first day of ECMO treatment and on the day of ECMO removal were collected and analyzed. All patients were treated with veno-venous ECMO after a median mechanical ventilation duration of 6.4±7.6 days. Among the 20 patients (52.6%) who were successfully weaned from ECMO, 16 patients (42.1%) survived to hospital discharge. Of the identified pre-ECMO factors, advanced age, a long duration of ventilation before ECMO, a higher Acute Physiology and Chronic Health Evaluation II (APACHE II) score, underlying lung disease, and pulmonary barotrauma prior to ECMO were associated with unsuccessful weaning from ECMO. Furthermore, multiple logistic regression analysis indicated that both barotrauma pre-ECMO and underlying lung disease were independent predictors of hospital mortality. In conclusion, for severe ARDS patients treated with ECMO, barotrauma prior to ECMO and underlying lung disease may be major predictors of ARDS prognosis based on multivariate analysis.

## Introduction

Despite years of focused researches and advancements in therapies, acute respiratory distress syndrome (ARDS) remains a fatal disease with a mortality rate of 40–46% [1, 2]. The new Berlin definition of ARDS guides physicians to the best treatment options based on illness severity and proposes ECMO as a valuable therapeutic option for patients with severe ARDS (PiO_2_/FiO_2_ below 100) to take over lung function and minimize ventilator-induced lung injury when conventional support fails [3].

ECMO is one of several terms used for an extracorporeal circuit that directly oxygenates and removes carbon dioxide from the blood. ECMO was first used successfully on an adult patient in 1971 [4]. Early studies in adults did not demonstrate a survival benefit from ECMO for severe acute respiratory failure and showed a mortality rate of approximately 60–80% [5–8]. However, technological advances and continued experience, particularly involving the use of ECMO for ARDS during the 2009 influenza A (H1N1) pandemic, generated widespread interest in ECMO techniques and increased the survival rate of ARDS patients treated with ECMO to 56%-70% between 2006 and 2010 [9–11]. Paden et al. showed that in the United States from 1996 to 2006, the use of ECMO remained steady at approximately 100 cases per year; however, in 2009, the use of ECMO dramatically increased to 400 cases per year [12]. Because this significant increase in the use of ECMO, which requires highly specialized staff and equipment, may increase resource utilization and hospital costs, early identification of mortality risk factors is needed [13]. However, outcome predictors for ARDS patients treated with ECMO remain unclear, and studies evaluating the mortality rate of severe ARDS adult patients undergoing ECMO based on the new Berlin definition of ARDS are scarce. The role and proper use of ECMO for ARDS patients have not been definitively established, despite the completion of the conventional ventilatory support vs extracorporeal membrane oxygenation for severe adult respiratory failure (CESAR) trial [14].

Therefore, the aim of our study was to review the use of ECMO in severe ARDS patients based on the new Berlin definition of ARDS. We analyzed the epidemiological characteristics, clinical features and predictors of survival among severe ARDS patients treated with ECMO in our center.

## Materials and Methods

### Ethics statement

This study was approved by the Institutional Research Ethics Board of the First Affiliated Hospital of Guangzhou Medical University (Permit No 2016–03), which waived the need for informed consent for the retrospective collection of demographic, physiological and hospital outcome data based on Chinese legislation. All patient records/data were anonymized and de-identified prior to analysis.

### Study design and patients

The First Affiliated Hospital of Guangzhou Medical University is a specialized acute-care university hospital. We reviewed the ECMO database, which identified all patients treated with ECMO between July 2009 and December 2014. We included all adult patients with a confirmed diagnosis of ARDS that was considered potentially reversible by the treating clinician based on the new Berlin definition of ARDS. Patients under 18 years of age were excluded from the study. ECMO therapy was indicated if patients exhibited a partial pressure of arterial oxygen (PaO_2_)/fraction of inspired oxygen (FiO_2_) ratio below 80 mmHg for at least 2 h with FiO_2_ of 1.0 and positive end expiratory pressure (PEEP)>5 cmH_2_O (We titrated PEEP according to ARDS network before ECMO support) or respiratory acidosis according to pH<7.20 despite the implementation of a lung-protective ventilation strategy (plateau pressure<30 cmH_2_O, tidal volume of 6 ml/kg).

### Data collection

Basic information was collected from our institution’s ECMO database for all patients. The following retrospective data were obtained: demographic data, such as age, sex, height and weight; primary diagnosis for ECMO implementation; chest radiographs; respiratory and hemodynamic parameters; ventilator settings; blood gas values; lactate levels; and APACHE II score, which was calculated according to data obtained prior to the initiation of ECMO. We evaluated the chest radiographs of patients to define pre-ECMO barotrauma events. Radiographic evidence of barotrauma included pneumothorax, pneumomediastinum, pneumatoceles, or subcutaneous emphysema. Because barotrauma in mechanically ventilated patients often presents as acute, life-threatening hypoxemia or hypotension, we reasoned that not all cases of barotrauma would have previously been documented on chest radiography before chest tube placement. As a consequence, we chose an inclusive definition of barotrauma that would capture most events [15]. We also collected data concerning ECMO management, including ECMO mode; cannulation; duration of ECMO support and complications; mechanical ventilation time before ECMO initiation and after ECMO support; interventions such as renal replacement therapy; dates of hospitalization, discharge from the hospital; and cause of death. The primary outcome for this study was the hospital mortality rate. The secondary end-point was the outcome of weaning from ECMO. Successfully weaned patients were defined as those who remained alive within 48 hours after weaning from ECMO.

### ECMO management

ECMO was initiated in our intensive care unit (ICU), which contains an established system with which to implement ECMO when needed. Percutaneous cannulation is performed by our ECMO team under general anesthesia in the ICU. The standard ECMO configuration for support of hypoxemic respiratory failure was veno-venous (femoro-jugular) ECMO. We used centrifugal pumps (Bioline, Maquet, Hirrlingen, Germany) at a flow rate of 3–5 L/min in all patients. Circuits were heparin-coated and composed of Quadrox PLS oxygenators (Bioline, Maquet, Hirrlingen, Germany) with HU 35 heater units (Maquet, Hirrlingen, Germany). Two circuit connectors were available between the pre- and post-oxygenators to provide renal replacement therapy via the ECMO circuit if required. Anticoagulation was maintained using continuous intravenous unfractionated heparin by targeting an activated clotting time of 160–180 s. The ventilator settings during ECMO were as follows: pressure control mode, PEEP 10–12 cmH_2_O, pressure above PEEP 12–15 cmH_2_O, plateau pressure 25–28 cmH_2_O, respiratory rate 16–18 breaths/min, and FiO_2_ adjusted to obtain an arterial O_2_ saturation of 90–95%; however, FiO_2_ was set to 1.0 on the oxygenator. 25/38 (65.8%) of the patients were curarized during the mechanical ventilation before the initiation of ECMO and all patients did not receive neuromuscular blocking agents during the ECMO support. ECMO was continued until lung recovery or until irreversible multiorgan failure leading to death. Patients were weaned from ECMO when the following criteria were met: after stopping gas flow to ECMO, a PaO_2_/FiO_2_ above 150 mmHg with PEEP<12 cmH_2_O, plateau pressure below 30 cmH_2_O and tidal volume of 5–7 ml/kg.

### Statistical analyses

Continuous variables are expressed as means ± standard deviation (SD) or medians with interquartile range, and categorical variables are expressed as percentages. Student’s t-test was applied to compare the means of continuous variables for normally distributed data; otherwise, the Mann-Whitney U test was employed. Categorical data were tested using the x^2^ test. Prognostic variables for mortality were analyzed by using the univariate logistic regression analyses, and variables with p <0.05 were used in multivariate logistic regression analyses with stepwise selection and the results were reported as odds ratios (ORs) and 95% confidence intervals (CIs). P<0.05 was considered statistically significant. Statistical analysis was performed using SPSS 12.0 software (SPSS, Inc., Chicago, IL, USA).

## Results

### Patient characteristics

**Fig 1:**
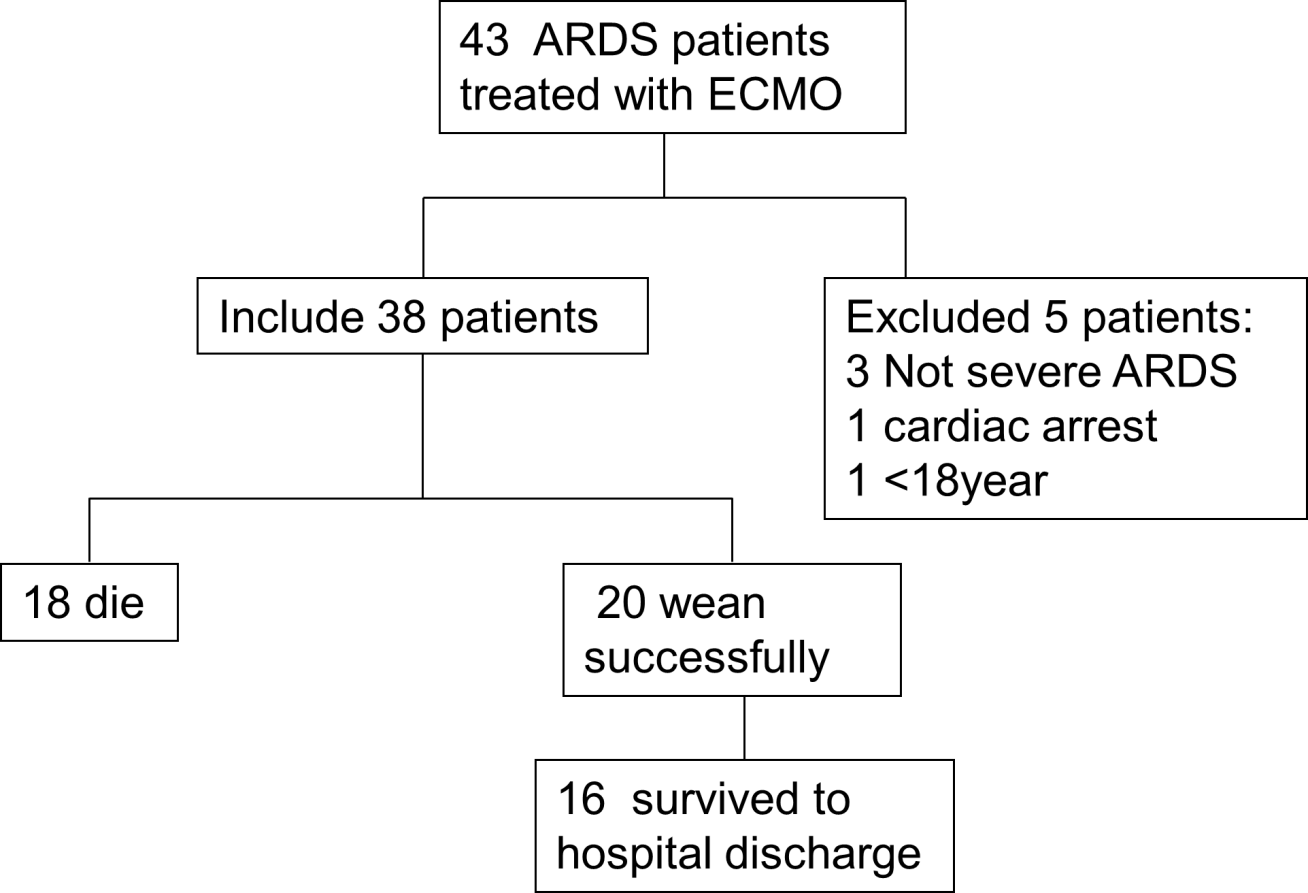
Flowchart and outcome of patients included in the study. ARDS, acute respiratory distress syndrome; ECMO, extracorporeal membrane oxygenation.

During the study period, 43 patients with severe respiratory failure received ECMO treatment, and 38 of these patients were analyzed. 1 patient under 18 years of age, 3 patients who did not have severe ARDS according to the new Berlin definition of ARDS, and 1 patient who experienced cardiac arrest were excluded from the study (Fig 1). As listed in Tables 1 and 2, 32 patients were male, and the mean age was 51.3±13.2 years (range 27–74 years). The average age of the successful ECMO group was lower than that of the unsuccessful ECMO group (49.9±13.7 vs 57.3±10.1 years, P<0.05), and the mean body mass index (BMI) was similar between the two groups (25.62±2.42 vs 26.36±2.95 kg/m^2^, P>0.05). Furthermore, 65% (13/20) of patients without underlying lung disease were successfully weaned from ECMO (P<0.05), but 9 patients who had a history of lung fibrosis died during ECMO. It was noted that of the 9 patients with interstitial lung disease which included 3 patients who were suspected to suffer from connective tissue disease involving the lung based on clinical diagnoses and 4 patients who were considered to suffer from idiopathic pulmonary fibrosis (IPF) and were awaiting lung transplantation, and 2 were diagnosed with interstitial lung disease of unknown origin. In addition, of the patients receiving ECMO, 91.67% (11/12) of those with pulmonary barotrauma prior to ECMO died while receiving ECMO. The main cause of ARDS was documented as infectious diseases (32/38), with 26 (81.25%) patients presenting with bacterial pneumonia, 3 with virus infection, and 3 with fungal pneumonia. The mean APACHE II score on first admission to the ICU was 21.34±3.98. The average APACHE II score of the non-survival group was higher than that of the survival group (22.18±3.49 vs 19.4±3.2, P<0.05).

**Table 1:** Baseline characteristics of the patients.

Characteristic	Total (n = 38)
Age (years)	51.39±13.27
Sex (male/female)	32 (84.2%)/6 (15.7%)
BMI (kg/m^2^)	25.73± 2.84
Underlying lung disease	
Healthy lungs	18 (47.3%)
COPD	6 (15.7%)
Lung fibrosis	9 (23.7%)
IPF	4 (10.5%)
CTD-relative fibrosis	3 (7.9%)
Unknown	2 (5.3%)
Lung carcinoma	2 (5.3%)
Post-lung transplantation	5 (13.2%)
Barotrauma	12 (31.6%)
Co-morbidities	
Hypertension	7 (18.4%)
Diabetes mellitus	5 (13.2%)
Renal insufficiency	19 (50.0%)
Infections	32 (84.2%)
Bacterial infection	26 (68.4%)
Viral pneumonia	3 (7.9%)
Fungal pneumonia	3 (7.9%)
APACHE II score	21.34±3.98

Continuous variables presented as means + SD, and categorical data are presented as numbers (%). BMI, body mass index; ARDS, acute respiratory distress syndrome; APACHE II, Acute Physiology and Chronic Health Evaluation II; CMV, cytomegalovirus; IPF, Idiopathic Pulmonary Fibrosis; CTD, Connective Tissue Disease.

**Table 2:** Baseline characteristics of patients who were successfully vs unsuccessfully weaned from ECMO and of survivors vs non- survivors.

Characteristic	Successful(n = 20)	Unsuccessful(n = 18)	*p-*value	Survivors (n = 16)	Non-survivors(n = 22)	*p-*value
Age (years)	49.9±13.7	57.3±10.1	0.039	50.±11.3	56.6±9.6	0.038
Sex (male/female)	16/4	16/2		12/4	20/2	
BMI (kg/m^2^)	25.6±2.4	26.3±2.9	0.398	25.5±2.5	26.2±2.7	0.39
Underlying lung disease						
Healthy lungs	13 (65.0%)	5 (27.8%)	0.024	11 (68.8%)	7 (31.8%)	0.027
COPD	4 (20.0%)	2 (11.1%)	0.384	2 (12.5%)	4 (18.2%)	0.498
Lung fibrosis	0	9 (50.0%)	<0.001	0	9 (40.9%)	<0.001
IPF	0	4 (22.2%)	0.041	0	4 (18.2%)	0.124
CTD-relative fibrosis	0	3 (16.7%)	0.158	0	3 (13.6%)	0.183
Unknown	0	2 (11.1%)	0.474	0	2 (65.0%)	0.329
Lung carcinoma	0	2 (11.1%)	0.218	0	2 (9.1%)	0.329
Post-lung transplantation	4 (20.0%)	1 (5.6%)	0.126	4 (25.0%)	1 (4.5%)	0.088
Barotrauma	1 (5.0%)	11 (61.1%)	<0.001	1 (6.3%)	11 (50.0%)	<0.001
Co-morbidities						
Hypertension (n)	5 (25.0%)	2 (11.1%)	0.249	5 (31.3%)	2 (9.1%)	0.108
Diabetes mellitus, % (n)	2 (10.0%)	3 (16.7%)	0.50	2 (12.5%)	3 (13.6%)	0.918
Renal insufficiency	8 (40.0%)	11 (61.1%)	0.165	6 (37.5%)	13 (59.1%)	0.325
Infections	15 (75.0%)	17 (94.4%)	0.234	13(81.3%)	19 (86.4%)	0.682
Bacterial infection	9 (45.0%)	17 (94.4%)	0.001	8 (50.0%)	18 (81.8%)	0.020
Viral pneumonia	3 (15.0%)	0	0.135	3 (18.8%)	0	0.066
Fungal pneumonia	3 (15.0%)	0	0.135	2 (12.5%)	1 (4.5%)	0.562
APACHE II score	19.7±4.079	23.17±3.015	0.006	19.4±3.2	22.18±3.49	0.019
PH	7.28±0.14	7.36±0.09	0.054	7.29±0.14	7.33±0.10	0.391
PaCO_2_ (mmHg)	65.76±26.78	57.12±18.44	0.263	57.87±13.9	64.49±28.2	0.394

Continuous variables presented as means + SD, and categorical data are presented as numbers (%). BMI, body mass index; ARDS, acute respiratory distress syndrome; APACHE II, Acute Physiology and Chronic Health Evaluation II; CMV, cytomegalovirus; IPF, Idiopathic Pulmonary Fibrosis; CTD, Connective Tissue Disease.

### Respiratory characteristics and ventilation variables before and after ECMO

As shown in Table 3, before ECMO, patients had severe respiratory failure despite advanced mechanical ventilator support, with a mean PaO_2_/FiO_2_ of 70.32±18.71 mmHg and a PEEP of 13.47±1.33 cmH_2_O. Notably, as shown in Table 4, early improvement of PaO_2_/FiO_2_ was significantly greater in ECMO survivors than in non-survivors after ECMO initiation (142.7±54.10 vs 107.4±23.36 mmHg, p<0.05) despite similar ventilator parameters and ECMO settings.

**Table 3:** Respiratory and ventilation characteristics of those who were successfully vs unsuccessfully weaned from ECMO in the 4 h before and during ECMO.

Characteristic		Total (n = 38)	Successful (n = 20)	Unsuccessful (n = 18)	*p*-value
Ventilation parameters					
PaO_2_/FiO_2_	Before	70.32±18.71	71.07±17.92	76.20±20.67	0.288
	After	119.8±43.12	135.5±51.39*p	102.4±27.49	0.048
PH	Before	7.32±0.126	7.28±0.14	7.36±0.096	0.054
	After	7.46±0.078	7.44±0.09	7.48±0.064	0.199
PaCO_2_ (mmHg)	Before	61.70±23.31	65.76±26.78	57.12±18.44	0.263
	After	39.3±7.02	39.84±7.42	38.70±6.71	0.626
PEEP (cmH_2_O)	Before	13.47±1.33	13.10±1.29	13.89±1.28	0.067
	After	10.71±1.41	10.35±1.53	11.11±1.18	0.098
Tidal volume (ml)	Before	411.6±26.05	412.5±28.81	410.6±23.38	0.822
	After	290.8±22.94	294.0±26.64	287.2±18.09	0.370
Plateau pressure	Before	27.32±1.165	27.55±1.98	28.39±1.24	0.132
	After	24.63±1.78	25.05±1.90	26.00±1.08	0.071
Respiratory rate	Before	27.69±5.18	26.85±4.59	29.06±5.50	0.130
	After	20.15±4.77	20.05±4.25*p	20.50±5.43	0.68
Heart rate	Before	110.5±18.4	113.2±20.07	107.7±16.56	0.372
	After	93.14±11.52	93.79±10.9*p	92.78±12.14	0.791
MAP	Before	69.76±4.53	70.47±5.19	68.98±3.65	0.318
	After	73.88±5.49	73.60±5.83	74.20±5.22	0.741
Other parameters					
WBC count, 10^9^/L	Before	14.46±5.67	13.19±6.03	15.46±4.58	0.27
	After	14.52±5.25	13.06±4.86	16.22±4.99	0.088
HCT	Before	30.14±5.73	30.35±6.62	29.89±4.52	0.806
	After	28.73±3.75	28.50±3.79	28.94±3.68	0.285
Hemoglobin	Before	103±21.0	101.3±24.26	105.4±17.11	0.550
	After	98.00±12.42	98.95±15.27	96.94±8.52	0.626
Lactate, mmol/L	Before	2.65 (0.78–10.65)	1.9 (0.78–5.99)	3.1 (1.68–10.65)	0.022
	After	2.15 (1.1–12)	2.12 (1.1–4.9)	2.10 (1.60–12)	0.429
Renal replacement therapy		12	5	7	0.45
Duration of ventilation before ECMO (days)		6.41±7.58	3.87±4.64	8.94±9.21	0.036
Duration of ECMO (days)		11.13±14.64	6.59±5.56	16.47±19.74	0.039
Duration of ventilation after ECMO (days)		16.84±15.59	16.73±17.17		

The data are expressed as n (%), medians (interquartile ranges), or means ± SD;

*p<0.05.

PaCO_2_, Partial pressure of carbon dioxide; PEEP, positive expiratory end pressure; MAP, mean arterial pressure; WBC, white blood cell; HCT, hematocrit.

**Table 4:** Respiratory and ventilation characteristics of survivors and non-survivors in the 4 h before and during ECMO.

Characteristic		Survivors (n = 16)	Non-survivors(n = 22)	*p*-value
Ventilation parameters				
PaO_2_/FiO_2_	Before	68.94±19.26	77.87±16.82	0.137
	After	142.7±54.10*	107.4±23.36	0.011
PH	Before	7.29±0.146	7.33±0.10	0.391
	After	7.43±0.07	7.47±0.074	0.083
PaCO_2_ (mmHg)	Before	57.87±13.91	64.49±28.28	0.394
	After	39.22±7.84	39.36±6.54	0.952
PEEP (cmH_2_O)	Before	13.19±1.377	13.68±1.29	0.26
	After	10.5±1.51	10.86±1.36	0.44
Tidal volume (ml)	Before	412.5±26.2	410.9±26.53	0.86
	After	291.9±24.01	290±22.28	0.81
Plateau pressure	Before	27.80±2.13	28.05±1.36	0.68
	After	25.38±1.89	25.45±1.62	0.89
Respiratory rate	Before	27.63±4.70	28.09±5.47	0.79
	After	20.05±4.60*	20.09±5.01	0.80
Heart rate	Before	113.8±17.6	108.3±18.61	0.37
	After	94.5±8.88*	92.41±12.78	0.58
MAP	Before	70.91±4.69	68.92±4.32	0.18
	After	74.29±6.13	73.59±5.09	0.70
Other parameters				
WBC count, 10^9^/L	Before	13.07±5.47	15.02±5.63	0.33
	After	13.42±5.36	15.08±4.76	0.36
HCT	Before	29.25±6.52	30.77±4.99	0.42
	After	28.13±4.06	29.41±3.44	0.41
Hemoglobin	Before	95.94±21.94	108.6±19.04	0.07
	After	96.69±14.24	98.95±11.16	0.58
Lactate, mmol/L	Before	1.9 (0.78–5.99)	2.80 (1.1–10.65)	0.09
	After	2.12 (1.4–4.9)	2.10 (1.1–12)	0.58
Renal replacement therapy		3	9	0.18
Duration of ventilation before ECMO (days)		3.08±3.89	8.59±8.69	0.023
Duration of ECMO (days)		5.73±4.91	15.57±17.88	0.040
Duration of ventilation after ECMO (days)		21.09±17.89		

The data are expressed as n (%), medians [interquartile ranges], or means ± SD;

*p<0.05.

PaCO_2_, Partial pressure of carbon dioxide; PEEP, positive expiratory end pressure; MAP, mean arterial pressure; WBC, white blood cell; HCT, hematocrit.

Most patients were mechanically ventilated for fewer than 7 days prior to the initiation of ECMO. The median time between intubation and ECMO cannulation was 6.41 (0.4–28) days. As shown in Table 4, compared with the surviving patients treated with ECMO, the non-survivors receiving ECMO experienced much longer mechanical ventilation durations before ECMO treatment (3.08±3.89 vs 8.59±8.69, p<0.05). In addition, the non-survivors displayed higher lactate levels [2.80 (1.1–10.65) vs 1.9 (0.78–5.99) mmol/L, P = 0.09]. During ECMO therapy, other characteristics were not significantly different between survivors and non-survivors. The median (range) duration of ECMO therapy was 5.73±4.91 days in survivors and 15.57±17.88 days in non-survivors. The duration of ECMO support was 56 days in one patient awaiting lung transplantation. In addition, the duration of mechanical ventilation after ECMO was 21.09±17.89 days in survivors. There was no significant difference in the creatinine or hemoglobin level or in the WBC count before ECMO between survivors and non-survivors. The duration of ECMO was longer in non-survivors than in survivors.

### Outcomes of patients and predictors of mortality

A total of 20 patients (52.63%) were successfully weaned from ECMO, and 16 patients (42.11%) survived to hospital discharge. The complications and outcomes of patients treated with ECMO are listed in Table 5. With respect to complications, hemorrhagic events occurred during ECMO in 16 patients (42.11%): gastrointestinal hemorrhage occurred in 9 patients, intrapulmonary hemorrhage occurred in 5 patients, intracerebral hemorrhage occurred in 1 patients and retroperitoneal hematoma in occurred in 1 patients; 1 of these patients required surgical treatment. Multiple organ failure associated with intractable respiratory failure was the most common cause of death; 18.18% of the patients died of severe infection, and 1 patient (4.5%) died of hemorrhagic complications.

**Table 5:** Outcomes of patients on ECMO support according to survival status.

Complications	Total (n = 38)	Survivors (n = 16)	Non-survivors(n = 22)	*p*-value
Major hemorrhagic complications	16	4	12	0.067
Gastrointestinal hemorrhage	9	5	4	0.45
Intrapulmonary hemorrhage	5	2	3	0.654
Intracerebral hemorrhage	1	0	1	0.579
Retroperitoneal hematoma	1	0	1	0.579
Deep venous thrombosis	4	1	3	0.433
Pulmonary embolism	3	0	3	0.183
Post-ECMO infection	5	1	4	0.286
Post-ECMO barotrauma	7	4	3	0.317
Acute kidney injury	11	4	7	0.466
**Cause of death**				
Multi-organ failure	12		12	
Irreversible respiratory failure	4		4	
Severe infection	4		4	
Intracerebral hemorrhage	1		1	
Others	1		1	

We further determined the relationship between patient characteristics and hospital mortality. Univariate analysis (Table 6) identified 4 variables as statistically significant prognostic factors for hospital mortality: age, duration of ventilation before ECMO, barotrauma pre-ECMO and underlying lung disease. We then included these 4 significant risk factors identified from univariate analysis in multivariate logistic analysis and Multivariate analysis (Table 6) showed that barotrauma pre-ECMO and underlying lung disease were significant and independent risk factors for hospital mortality, whereas Age (p = 0.567) and Duration of ventilation before ECMO (p = 0.117) were not significant.

**Table 6:** Multivariate logistic regression analysis: independent predictors of mortality.

	Univariate logistic regression		Multivariate logistic regression		
Variable	OR	*p*	OR	95% CI	*p*
Age	4.444	0.034	1.892	0.213–168.22	0.567
Duration of ventilation before ECMO	1.190	0.044	1.232	0.949–1.599	0.117
ECMO duration	1.117	0.111			
APACHE II score before ECMO	1.203	0.082			
PaO_2_/FiO_2_ before ECMO	1.013	0.420			
Lactate before ECMO	1.304	0.243			
WBC before ECMO	1.073	0.322			
Hemoglobin before ECMO	1.0320	0.074			
Barotrauma pre-ECMO	26.25	0.004	34.176	2.193–532.497	0.012
Underlying lung disease	14.733	0.001	12.213	1.220–122.242	0.033

OR: odds ratio

## Discussion

ECMO has been controversial because of its association with serious complications and poor outcomes over the last several years; however, advances in extracorporeal technology have renewed interest based on accumulating new evidence [16]. To date, most studies examining the rates and causes of death among critically ill patients, such as those with severe myocardial dysfunction and life-threatening respiratory failure, focused on the time point after the initiation of ECMO. In a large multicenter database of 1,473 adult patients supported with ECMO during respiratory failure, the rate of survival to hospital discharge was 50% [17]. The results of our study show that the mortality rate of adult patients suffering from severe ARDS undergoing ECMO is 57.89%. Studies evaluating the mortality rate of severe ARDS adult patients undergoing ECMO based on the new Berlin definition of ARDS are scarce. Several authors have reported the successful use of ECMO on patients with influenza A (H1N1) virus-induced ARDS infection-associated severe respiratory failure in Australian and New Zealand ICUs, resulting in a mortality rate varying from 21 to 33% [10, 18]. In the CESAR trial, 63% of patients treated with ECMO survived, and this result demonstrated that ECMO produced favorable outcomes [14]. Most studies have shown that application of ECMO results in encouraging survival rates.

This is the first article to review the use of ECMO on severe ARDS patients admitted to the ICU in China based on the new Berlin definition of ARDS and to identify predictors of mortality among severe ARDS patients supported with ECMO. Compared with previous studies, the present study reported a higher mortality rate. Several factors may have accounted for the higher mortality rate at our center. First and most importantly, the present study reviewed the use of ECMO on severe ARDS patients according to the new Berlin definition of ARDS. A PiO_2_/FiO_2_>100 is an exclusion criterion for ECMO treatment; therefore, some ARDS patients with a PiO_2_/FiO_2_>100 and high carbon dioxide levels were excluded from this study. The patients enrolled in our study who were supported with ECMO may have been very sick and may have had refractory hypoxia [3]. Additionally, our center is considered as a regional reference center for the treatment of the most severe cases, and other centers would likely not use ECMO at the same rate on such high-risk patients; this difference could explain the high ARDS severity observed in our patients [19]. Second, compared with other studies, the present study reported a longer mean duration of ventilation before ECMO, and this result likely contributed to the high mortality rate. As reported previously, the ventilation time before ECMO is related to the risk of mortality: the longer the ventilation time, the higher the mortality rate [20]. Finally, most patients enrolled in this study had pneumonia associated with severe sepsis, and many of these patients required renal replacement therapy during ECMO support; these conditions may also have contributed to the high mortality rate observed in this study [21]. The mortality rate is most likely strongly infiuenced by characteristics that vary among centers [22].

Another objective of this study was to identify early prognostic factors of successful weaning from ECMO and mortality among severe ARDS patients treated with ECMO to help clinicians decide whether to treat patients with ECMO. We found that age, duration of ventilation before ECMO, underlying lung disease, and barotrauma prior to ECMO affected the hospital mortality rate of ARDS patients treated with ECMO. Furthermore, we observed that the average age of the survival group was lower than that of the non-survival group. Specifically, 53.8% of the patients less than 50 years old had a markedly good prognosis without severe disability, whereas most patients over 50 years of age did not survive when treated with ECMO. This result suggests that ECMO should be considered for young patients, even if they have other contraindications, and this conclusion is in agreement with many other studies [19–21, 23]. Based on the present results, in addition to age, the duration of mechanical ventilation before ECMO was associated with mortality. According to the established protocol [12], a 7-day duration of ventilation is the cut-off point: a ventilation time>7 days is an exclusion criterion for ECMO treatment. Notably, in our study, most of the patients with barotrauma before ECMO were not successfully weaned from ECMO. This observation suggests that barotrauma prior to ECMO is associated with death, and this relationship between barotrauma and mechanical ventilation may explain why the duration of mechanical ventilation before ECMO was also associated with poor prognosis. However, in our study, 3 patients with ventilation times before ECMO of 9, 15, and 17 days were successfully weaned from ECMO and survived to hospital discharge. This result suggests that ECMO support could be initiated on patients who had been mechanically ventilated for more than 7 days if there was no barotrauma prior to ECMO. It may be appropriate to focus on the implementation of a protective ventilation strategy before ECMO. Further studies are necessary to clearly address this issue. In addition, in our study population, there were 9 ARDS patients with lung fibrosis, including fibrosis in the primary and secondary stages, such as connective tissue diseases; ECMO failed in these patients. This result suggests that physicians should select appropriate candidates for ECMO among severe ARDS patients. Patients with potentially irreversible underlying lung diseases such as connective tissue diseases should not be recommended for ECMO unless they are awaiting a lung transplant. Although the severity of hypoxemia before ECMO was not different between ECMO survivors and non-survivors, we observed a greater improvement in PaO_2_ among survivors than among non-survivors after ECMO initiation, and this improvement was associated with the implementation of protective ventilation.

Complications of ECMO, such as bleeding, remain a clinically significant issue [24]. Hemorrhagic events occurred in 16 patients (42.11%) undergoing ECMO in our study. Non-survivors displayed a higher rate of complications, including hemorrhage, deep venous thrombosis, pulmonary embolism, infection and renal complications. Most patients experienced at least one complication attributed to ECMO, e.g., brain death, cerebral infarction and seizures, thromboembolism, and circuit clots [4]. Other centers have found similarly high complication rates [19–21, 23]. Notably, one patient who suffered from intrapulmonary hemorrhage was successfully treated via endoscopic hemostasis and survived to discharge. Nevertheless, the most common causes of death among ARDS patients are related to multiple organ failure associated with intractable respiratory failure and severe sepsis. As shown previously, the lactate levels of the non-survival group were higher than those of the survival group; this result suggests that ECMO may be less useful for severe ARDS patients with septic shock, greater cardiac output, and impaired peripheral oxygen extraction when their pulmonary gas-exchange capacity is severely impaired [13, 25, 26]. Whether ECMO is an appropriate therapy for septic adults is still controversial [27].

Importantly, ECMO assistance was successfully used as a bridge to lung transplantation in one patient with pulmonary fibrosis in our study. The patient survived to discharge without disability and had returned to work by the end of the study. Our center has begun using ECMO for critically ill adults as a bridge to lung transplantation, even though the currently available data are limited. However, the use of ECMO while awaiting lung transplantation has been very promising in some patients with reversible lung disease [28, 29].

Another important point to discuss is the ECMO team. It is essential to recognize the crucial nature of the collaborative effort of physicians, respiratory therapists and nurses to manage patients using complex technology safely and effectively in the ICU [30]. ECMO should be performed at centers with high case volumes, established protocols, and clinicians who are experienced in its use [31].

Several limitations of this study should be acknowledged. First, this was a retrospective study performed at a single medical center, and this design limits the generalizability of these findings. Second, the number of included patients was small, and we failed to evaluate the long-term outcome of our patients, particularly in relation to the degree of pulmonary dysfunction and quality of life; this lack of data limited the conclusions that could be drawn. Future work in this area, where possible, should include a larger number of subjects and a control group not receiving ECMO from the same population. One strength of the present study is that it only includes patients with severe ARDS according to the New Berlin definition. Previous studies have frequently mixed patients with ARDS and those with cardiogenic shock, but these diseases are likely to affect different populations and to have different prognostic factors [32].

In conclusion, our results revealed a hospital mortality rate of 57.89% among severe ARDS patients. Our data also demonstrated that advanced age, a long duration of ventilation before ECMO, underlying lung disease and barotrauma prior to ECMO affected the mortality rate of ARDS patients being treated with ECMO. Barotrauma prior to ECMO and underlying lung disease were independent prognostic factors for survival to hospital discharge among ARDS patients treated with ECMO based on multivariate analysis. These results might help physicians select appropriate candidates for ECMO among severe ARDS patients. Accordingly, our findings indicate that ECMO can be used as an alternative therapy for severe ARDS patients when conventional ventilation fails. However, additional studies should be conducted to further define the indications for ECMO use in severe ARDS patients.
